# Pilot study of *Clostridioides difficile* infection (CDI) in hospitals, Italy, September to December 2022

**DOI:** 10.2807/1560-7917.ES.2025.30.1.2400206

**Published:** 2025-01-09

**Authors:** Patrizia Spigaglia, Fabrizio Barbanti, Enrico Maria Criscuolo, Fortunato D’Ancona, on behalf of the Italian CDI Surveillance Pilot working group

**Affiliations:** 1 Department of Infectious Diseases, Istituto Superiore di Sanità, Rome, Italy; 2 The members of this group are listed under Acknowledgements

**Keywords:** *Clostridioides difficile*, CDI, surveillance, epidemiology, microbiology, typing

## Abstract

**Background:**

*Clostridioides difficile* infection (CDI) is a severe infection that needs to be monitored. This infection predominantly occurs in hospitalised patients after antimicrobial treatment, with high mortality in elderly patients.

**Aim:**

We aimed at estimating the incidence of CDI in Italian hospitals over 4 months in 2022.

**Methods:**

We estimated incidences of hospital-acquired CDI (HA-CDI), community or unknown CDI (CA/UA-CDI), recurrent CDI and overall CDI in 25 Italian hospitals, characterised *C. difficile* isolates using PCR ribotyping, analysed them for toxin genes and susceptibility to antimicrobials.

**Results:**

*Clostridioides difficile* was detected in 9.7% (655/6,722) of samples from 550 patients, 18 patients died of CDI. The mean overall CDI incidence was 5.0 cases per 10,000 patient days (range: 0.7–11.9). For HA-CDI, mean incidence was 3.7 (range: 0.7–9.2), for CA/UA-CDI 0.8 (range: 0.0–3.2) and for recurrent CDI 0.5 (range: 0.0–3.4). Most patients were female (n = 295; 53.6%), aged ≥ 65 years (n = 422; 76.7%) and previously hospitalised (n = 275; 50.0%). Of the 270 culturable isolates, 267 (98.9%) had toxin A and B genes and 51 (18.9%) the binary toxin genes. Of the 55 PCR ribotypes (RTs) identified, RT 018 (n = 56; 20.7%) and RT 607 (n = 23; 8.5%) were the most common, RT 607 in the northern (p < 0.0001) and RT 018 in the central (p < 0.0001) regions of Italy. Most isolates (n = 158; 58.5%) were antimicrobial-resistant and 119 (44.1%) were multidrug-resistant (MDR).

**Conclusion:**

Highly virulent and MDR *C. difficile* types are circulating in Italian hospitals which highlights the need of robust surveillance and stringent prevention and control measures.

**Key public health message**

**What did you want to address in this study and why?**

*Clostridioides difficile* infection (CDI) is common in hospitalised patients after antimicrobial treatment, causing diarrhoea and a severe inflammation of the intestine. The infection can be difficult to treat and life-threatening. We wanted to know how common CDI is in patients hospitalised in Italy, if *C. difficile* strains are resistant to antimicrobials and how this infection could be monitored.

**What have we learnt from this study?**

Over a period of 4 months, we detected *C. difficile* in samples in from 550 patients in 25 hospitals in Italy. More than half of the patients were female, aged ≥ 65 years or previously hospitalised. More than half of the 270 isolates causing infection were resistant to antimicrobials, 119 were resistant to three or more classes of antimicrobials (MDR), and almost all were highly virulent.

**What are the implications of your findings for public health?**

The results of the study indicate that a national surveillance of this infection is necessary in Italy. International cooperation is also necessary to monitor the emergence of new *C. difficile* types that are resistant to antimicrobials, spread fast and cause more severe disease.

## Introduction

*Clostridioides difficile* is the leading cause of antimicrobial-associated diarrhoea in the hospital environment [1]. Symptoms of a clinical *C. difficile* infection (CDI) range from slightly loose stools or diarrhoea to more severe manifestations (pseudomembranous colitis and toxic megacolon) that may be life-threatening [2]. After the emergence and the worldwide spread of the PCR ribotype (RT) 027 at the beginning of this millennium, other RTs with characteristics of high virulence, recurrences and multidrug resistance (MDR) have emerged at local or international levels [3,4]. The global incidence of CDI is estimated to 49.36 cases per 100,000 population per year, with varying geographic rates [5].

Periodic or continuous standardised monitoring of CDI allows observation of epidemiological changes to prevent and control this infection [6]. Due to the lack of standardised CDI surveillance in the European Union/European Economic Area (EU/EEA) countries, European Centre for Disease Prevention and Control (ECDC) launched the European *Clostridium difficile* Surveillance Network (ECDIS-Net) project and a survey on CDI in acute care hospitals to encourage and support European countries in setting up and activating a national CDI surveillance [7-10]. With the aim to harmonise the CDI surveillance in the EU/EEA countries, ECDC has also addressed different aspects of this infection such as case definitions, criteria for inclusion and exclusion of CDI cases or patients, methodologies and data collection tools required, in a surveillance protocol published in 2019 [11].

Different from some other European countries, Italy does not have an active national surveillance of CDI. So far, Italian data on CDI have been partially retrieved from national or international retrospective studies. Among the most recent is the Longitudinal European *Clostridium difficile* Infection Diagnosis surveillance study (LuCID) which 2014–2015 investigated the variability in *C. difficile* sampling and CDI incidence rates in three European countries, including 20 Italian hospitals. A study of the Italian Scientific Society of Hospital Internal Medicine (FADOI) evaluated the rate, the diagnostic work-up and the outcome of CDI in the medical wards of 40 Italian hospitals 2013–2014 [12,13].

Between 2020 and 2022, the Italian Ministry of Health funded a project to support the surveillance of the healthcare-associated infections (HAI), according to the suggestions of the Italian national plan to combat antibiotic resistance (Piano Nazionale di Contrasto all’Antibiotico-Resistenza - PNCAR; https://www.ccm-network.it/progetto.jsp?id=node/2042&idP=740; https://www.epicentro.iss.it/sorveglianza-ica/informazioni-generali). One of the main objectives of the project was to lay the foundations for CDI surveillance in Italy. With this aim, we performed a pilot study on surveillance of CDI in Italian hospitals.

## Methods

### Design of the pilot study and settings

In the guideline, ECDC proposes three options for CDI surveillance: (i) minimal surveillance, i.e. collection of aggregated numerator and denominator data; (ii) light surveillance, i.e. collection of case-based and aggregated denominator data; (iii) enhanced surveillance, that has the same characteristics as the light surveillance, but also includes collection of microbiological data, i.e. analysis of at least five isolates from consecutive CDI cases from each participating hospital during the surveillance period [11]. We used the option of enhanced surveillance in our pilot study performed between September and December 2022.

The Italian healthcare system operates across different political and administrative levels: the national central level administered by the Ministry of Health, the 19 regions and two autonomous provinces and the local level. The Italian hospital levels are three: primary (catchment area of 80,000–150,000 inhabitants), secondary (catchment area of 150,000–300,000 inhabitants, with an emergency department and specialties) and tertiary (catchment area of 600,000–1,200,000 inhabitants, with an emergency department and many specialties, often university hospitals or associated with a university).

The Italian Public Health Institute, Istituto Superiore di Sanità (ISS), as the national coordinator of the present pilot study, informed the reference person of each Italian region and autonomous province about the study, asking them to recruit hospitals. The reference persons invited via e-email several hospitals and sent to the ISS names of hospitals consenting to participate in the study, without any incentives. The coverage of the included hospitals was calculated as a proportion of patients per day in these hospitals in 2022 of the total number of patient days (pd) in all hospitals in the same area in the same period. The number of pd per hospital was obtained from the national hospital discharge records database of ISS.

### Case definitions

The inclusion criteria (for hospital wards and patients) and the case definitions for CDI, CDI recurrence, origin of infection, healthcare-associated CDI (HA-CDI), community or unknown CDI (CA/UA-CDI) as recommended by ECDC, were used in the study [11]. The definitions are described in Supplementary File S1. In Italy, the Italian Multidisciplinary Society for the Prevention of Infections in Healthcare Organizations (SIMPIOS) recommends that samples of diarrhoeal stools (Bristol scale 5-7) should be tested for *C. difficile,* and that the investigation should be repeated in case of a first negative result for *C. difficile* and a strong clinical suspicion of CDI [14].

An operative protocol and a short guide for using the software HelicsWin.Net (HWN) for data collection, available free of charge on the ECDC website (https://www.ecdc.europa.eu/en/publications-data/helicswinnet-hwn), were translated to Italian by the ISS to facilitate data entry by the healthcare operators involved in the pilot study. In addition, the ISS directly provided support to operators via email or telephone contact, if required.

### Data collection

Each participating hospital had a numeric identification code provided by the Italian Ministry of Health. In each hospital, the data collected for each CDI case and the *C. difficile* isolate from that case were referred to a sequential numeric code generated by the HWN software. The hospital and the case-based data, entered into the respective sections of the HWN software, were exported by each hospital into a Microsoft Access file for the transmission to the reference person of the respective region or autonomous province and to the ISS.

In the ISS, the HWN codes were pseudonymised. Consequently, the data and the *C. difficile* isolate collected from each CDI case were also pseudonymised. During the pilot study, a maximum of 40 *C. difficile* isolates (10 per month) from each hospital were collected from different consecutive CDI cases and sent to the ISS. After the analysis, the ISS recorded the microbiological data into the respective section of the HWN software.

Epidemiological and microbiological data collected during the study were analysed and summarised by the ISS in a final report that was sent to the participating Italian regions or autonomous provinces and the Italian Ministry of Health. Finally, all data collected were uploaded to the European Surveillance System (TESSy; https://www.ecdc.europa.eu/en/publications-data/european-surveillance-system-tessy).

### Molecular characterisation of *Clostridioides difficile* isolates

All *C. difficile* isolates received at the ISS were inoculated onto selective CHROMID *C. difficile* plates (bioMérieux, Marcy l’Étoile, France). After 48 h of incubation in an anaerobic cabinet (90% N_2_, 5% CO_2_ and 5% H_2_), one colony of *C. difficile* was re-inoculated onto blood agar plates supplemented with 5% sheep blood, 5 mg/L haemin and 0.5 mg/L vitamin K and after 24 h of incubation in anaerobic atmosphere, several colonies were stored in a cryotube at −80°C for subsequent analysis.

Bacterial DNA was extracted by suspending several fresh colonies of *C. difficile* in 100 μL of 5% w/v Chelex-100 resin (Bio-Rad, Hertfordshire, the United Kingdom (UK)) in molecular grade H_2_O. The bacterial suspension was heated at 100°C for 10 min and the lysate was centrifuged for 3 min at 16,000 g. The supernatant obtained was collected and the DNA concentration was adjusted to 100 ng/μL.

All *C. difficile* isolates were analysed for the presence of the genes coding for toxins A and B (*tcdA* and *tcdB*) and for the binary toxin CDT genes (*cdtA* and *cdtB*) by a multiplex PCR assay, as recommended by the Standard Operating Procedures (SOPs) of ECDC [15].

All *C. difficile* isolates were characterised using the capillary PCR ribotyping method and the RTs were identified using the free web-database WEBRIBO (http://webribo.ages.at), as previously described [15,16].

### Antimicrobial susceptibility testing

As suggested by ECDC [11], we evaluated the susceptibility of *C. difficile* isolates to metronidazole (MTZ) and vancomycin (VAN), used in the treatment of CDI, and moxifloxacin (MXF), recently proposed as a considerable risk factor for CDI. In addition, we tested erythromycin (ERY) and clindamycin (CLI), which have been associated with CDI, and rifampicin (RIF), widely used in Italy for decades and considered as an adjunctive therapy to reduce the recurrence of CDI [17]. The minimum inhibitory concentrations (MIC) for these antimicrobials were estimated by agar dilution method: the isolates were inoculated onto pre-reduced *Brucella* agar (Thermo Fisher, Waltham, the United States (US)) plates supplemented with 5 mg/L haemin, 1 mg/L vitamin K1 and 5% defibrinated sheep red blood cells, as recommended by the Clinical and Laboratory Standards Institute (CLSI) [18]. We recorded the MIC values after 24 h (48 h for CLI) of incubation in anaerobic conditions.

The breakpoint used for MXF, ERY and CLI was 8 mg/L, while the breakpoint for RIF was 4 mg/L in accordance with the CLSI interpretive categories approved for *Staphylococcus aureus* [18,19]. Reduced susceptibility to MTZ and VAN was defined as MIC > 2 mg/L, according to the epidemiological cut-off values suggested by the European Committee on Antimicrobial Susceptibility Testing (EUCAST) [20].

An isolate had a resistant phenotype if it showed resistance to one or two classes of the antimicrobials tested, while it had a MDR phenotype if it was resistant to three or more classes of the antimicrobial classes tested.

### Statistical analyses

Statistical analyses were performed using the two-tailed Fisher’s exact test, and a p value of < 0.05 was considered statistically significant. Analyses were performed using GraphPad Prism software (GraphPad Software, La Jolla, US), including the following variables: geographic area of the hospitals, type of hospital wards, sex, age, patient outcome, type of CDI cases (CA, HA, UA, recurrent), RT, resistance phenotype and presence of binary toxin gene of isolates.

## Results

### Participating hospitals

**Figure 1:**
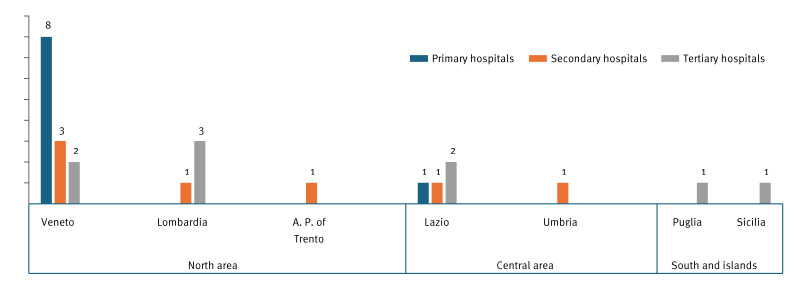
Geographic location of hospitals participating in a pilot study on *Clostridioides difficile* infection (CDI), Italy, September–December 2022 (n = 25 hospitals)

In total, 25 hospitals from six Italian regions and one autonomous province participated in the study: nine primary hospitals, seven secondary hospitals and nine tertiary hospitals (Figure 1, Table). Eighteen hospitals were from the north area, five from the central area and two from the south and the islands. The hospitals had altogether 14,582 beds. These hospitals covered 7.3% (annual pd of hospitals participating in the study (n = 3,626,911)/annual pd of all hospitals located in the participating regions and autonomous provinces (n = 49,709,743)) of all hospitals located in the seven regions or autonomous provinces participating in the study (range: 4.8%–34.3%).

A.P.: autonomous province.

The number of hospitals participating is given above the bars and the names of the participating regions or autonomous provinces are indicated in the boxes.

**Table:** Results of a pilot study on *Clostridioides difficile* infection (CDI) in hospitals, Italy, September–December 2022 (n = 25 hospitals)

Characteristics	Primary hospital (n = 9)^a^			Secondary hospital (n = 7)^a^			Tertiary hospital (n = 9)^a^			Total	
	n	Range		n	Range		n	Range			
Geographic area											
North	8			5			5			18	
Centre	1			2			2			5	
South and islands	0			0			2			2	
Capacity											
Number of patient days	137,384^b^			286,337^b^			636,887^b^			1,060,608^b^	
Number of beds	2,039	48–400		3,812	148–965		8,731	460–1,473		14,582	
Number of samples tested	969	0–394		1,538	37–709		4,215	100–1,192		6,722	
*Clostridioides difficile* detected	n	Range	%	n	Range	%	n	Range	%	n	%
Number of detections	80	0–42	8.3	200	3–80	13.0	375	16–70	8.9	655	9.7
Diagnostic algorithms applied											
ESCMID^c^	1			4			4			9	
Other algorithms	2			3			5			10	
Unknown	6			0			0			6	
Cases	n = 44			n = 206			n = 300			n = 550	
Source of CDI	n	%		n	%		n	%		n	%
Healthcare-associated	28	63.6		151	73.3		233	77.7		412	74.9
Community-associated	11	25.0		35	17.0		44	14.7		90	16.4
Recurrences	5	11.4		20	9.7		23	7.7		48	8.7
Age	Time	Range		Time	Range		Time	Range		Time	
Average age of patients (years)	76.2	6–94		73.3	1–98		70.3	0.2–105		71.9	
Hospital stay											
Average length (days)^d^	20.7	4–105		25.7	1–107		31.4	2–117		28.1	
Sex											
Male	17	38.6		91	44.2		146	48.7		254	46.2
Female	27	61.4		115	55.8		153	51.0		295	53.6
Unknown	0	0.0		0	0.0		1	0.3		1	0.2
Age of patients with CDI											
≤ 18 years	1	2.3		3	1.5		19	6.3		23	4.2
19–64 years	6	13.6		42	20.4		57	19.0		105	19.1
≥ 65 years	37	84.1		161	78.2		224	74.7		422	76.7
Previous hospital stay											
Yes	24	54.5		112	54.4		139	46.3		275	50.0
No	16	36.4		82	39.8		89	29.7		187	34.0
Unknown	4	9.1		12	5.8		72	24.0		88	16.0
Symptoms of CDI on admission											
Yes	18	40.9		62	30.1		61	20.3		141	25.6
No	19	43.2		127	61.7		167	55.7		313	56.9
Unknown	7	15.9		17	8.3		72	24.0		96	17.5
Patient treated for CDI											
Yes	7	15.9		149	72.3		192	64.0		348	63.3
No	1	2.3		12	5.8		24	8.0		37	6.7
Unknown	36	81.8		45	21.8		84	28.0		165	30.0
Deaths of patients with CDI (n = 73)											
CDI-related^e^	1			8			9			18	
Total	11			27			35			73	

CA/UA-CDI: community-associated or unknown-associated CDI; CDI: *Clostridioides difficile* infection; ESCMID: The European Society of Clinical Microbiology and Infectious Diseases; HA-CDI: hospital-associated CDI.

^a^ Primary hospital: catchment area of 80,000–150,000 inhabitants; secondary hospital: catchment area of 150,000–300,000 inhabitants, with an emergency department and specialties; tertiary hospital: catchment area of 600,000–1,200,000 inhabitants, with an emergency department and many specialties, often university hospital or associated with a university.

^b^ Data from 21 hospitals: primary hospitals: (n = 8); secondary hospitals (n = 6); tertiary hospitals (n = 7).

^c^ Two-step algorithms, including a test to detect *Clostridioides difficile* free toxins [15].

^d^ Data reported for 396 of 550 patients with CDI (this information was not available for all patients of the 25 participating hospitals).

^e^ Definitely (an infection is considered certainly caused by *Clostridioides difficile*) or possibly related to CDI. Hospitals entered this information in the HelicsWin.net (https://www.ecdc.europa.eu/en/publications-data/helicswinnet-hwn).

### Patients with *Clostridioides difficile* infection (CDI)

*Clostridioides difficile* was detected from 655 (9.7%) of the 6,722 samples tested (Table). Of the 655 *C. difficile*-positive samples, 550 were from confirmed CDI cases: 412 (74.9%) were HA-CDIs, 90 (16.4%) CA/UA-CDIs and 48 (8.7%) were recurrences.

**Figure 2:**
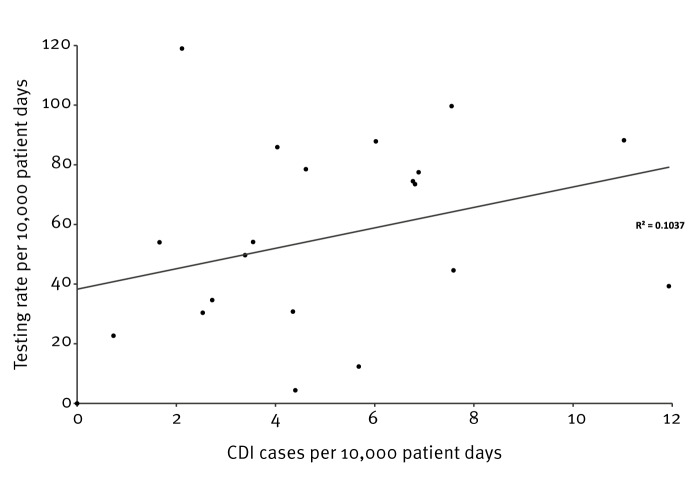
Correlation between testing rate and overall *Clostridioides difficile* infection (CDI) incidence rate per 10,000 patient days in hospitals participating in a pilot study on *Clostridioides difficile* infection (CDI), Italy, September–December 2022 (n = 25 hospitals)^a^

**Figure 3:**
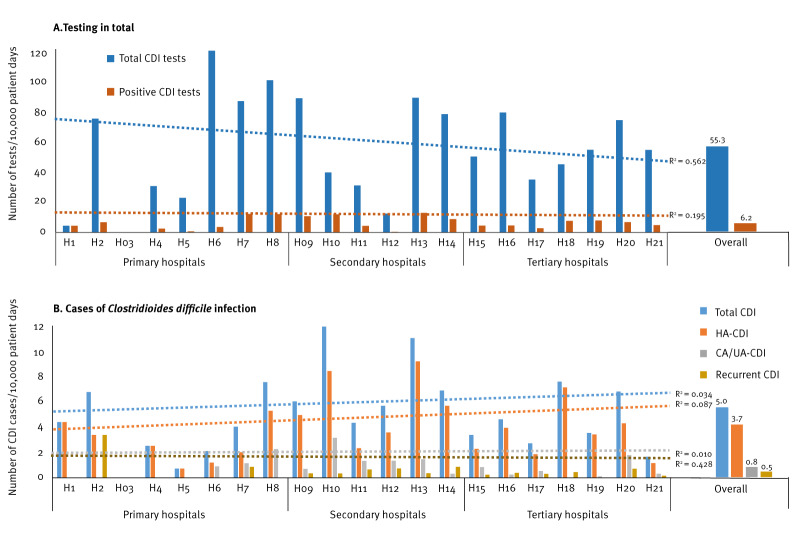
*Clostridioides difficile* testing per 10,000 patient days in hospitals in a pilot study on *Clostridioides difficile* infection (CDI), by hospital level, Italy, September–December 2022 (n = 25 hospitals)^a^

CDI: *Clostridioides* *difficile* infection; HA-CDI: hospital-associated CDI; CA/UA-CDI: community-associated or unknown.

^a^ Data were obtained from 21 of the 25 hospitals participating in the pilot study.

Twenty-one of the 25 participating hospitals reported the number of pd in the HWN software. The number of CDI cases was proportional to the number of *C. difficile* tests performed (Figure 2). A mean testing rate was 55.3 tests per 10,000 pd (range: 0–119 tests/10,000 pd) and a mean rate of CDI-positivity was 6.2 detections per 10,000 pd (range: 0–12.9 detections/10,000 pd) (Figure 3A).

CDI: *Clostridioides difficile* infection; R^2^ : coefficient of determination.

^a^ Data were obtained from 21 of the 25 hospitals participating in the pilot study.

In Panel A, the overall columns represent the mean overall CDI testing rate (in blue) and the mean *C. difficile* positive rate (in orange).

In Panel B, the overall columns represent the mean overall CDI incidence rate (in blue), the mean HA-CDI incidence rate (in orange), the mean CA/UA-CDI incidence rate (in grey) and the recurrent CDI incidence rate (in yellow). The dotted lines represent trend lines of CDI tests (A) and CDI cases (B) for the different hospital levels.

Primary hospital: catchment area of 80,000–150,000 inhabitants; secondary hospital: catchment area of 150,000–300,000 inhabitants, with an emergency department and specialties; tertiary hospital: catchment area of 600,000–1,200,000 inhabitants, with an emergency department and many specialties, often university hospital or associated with a university.

Primary hospitals performed more *C. difficile* tests than the other hospitals (Figure 3A), while the number of CDI cases varied slightly among the hospital levels (Figure 3B).

Nine hospitals adopted the testing algorithms recommended by the European Society of Clinical Microbiology and Infectious Diseases (ESCMID) [15], 10 used other algorithms and six hospitals did not give information about the algorithms used (Table). The ESCMID algorithms were used in one primary, four secondary and four tertiary hospitals, located in four of the seven regions or autonomous provinces, as presented in Supplementary Table S1. The overall CDI rate in hospitals using an ESCMID algorithm ranged between 4.1 and 11.9 cases per 10,000 pd, while in hospitals using other diagnostic algorithms it ranged between 1.8 and 6.9 cases per 10,000 pd.

The CDI rates varied between the 21 hospitals providing data (Figure 3B). The mean rate for the overall CDI was 5.0 cases per 10,000 pd (range: 0.7–11.9 cases/10,000 pd): HA-CDI was 3.7 cases per 10,000 pd (range: 0.7–9.2 cases/10,000 pd), CA/UA-CDI was 0.8 per 10,000 pd (range: 0–3.2 cases/10,000 pd) and recurrent CDI was 0.5 cases per 10,000 pd (range: 0–3.4 cases/10,000 pd) (Figure 3B).

Of the patients with CDI, 295 (53.6%) were female, 422 (76.7%) were aged ≥ 65 years, 275 (50.0%) were previously hospitalised and 313 (56.9%) were asymptomatic on admission (Table). Most of these patients (63.3%) were treated for CDI, with an average hospital stay of 28.1 days. In total, 13.3% of patients with CDI died, and CDI was associated with 18 (3.3%) deaths.

**Figure 4:**
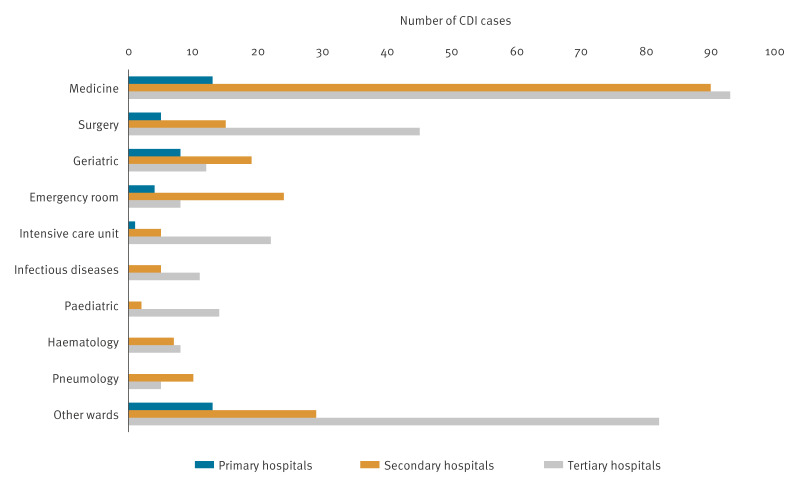
Patients with *Clostridioides difficile* infection (CDI) in hospitals participating in a pilot study on *Clostridioides difficile* infection (CDI), by ward and hospital level, Italy, September–December 2022 (n =550)

Patients with CDI were from the general medicine (n = 198; 36%), surgery (n = 61; 11%) and geriatric (n = 38; 7%) wards, independent of the hospital level (Figure 4).

CDI: *Clostridioides difficile* infection.

Primary hospital: catchment area of 80,000–150,000 inhabitants; secondary hospital: catchment area of 150,000–300,000 inhabitants, with an emergency department and specialties; tertiary hospital: catchment area of 600,000–1,200,000 inhabitants, with an emergency department and many specialties, often university hospital or associated with a university.

### Typing and microbiological data

**Figure 5:**
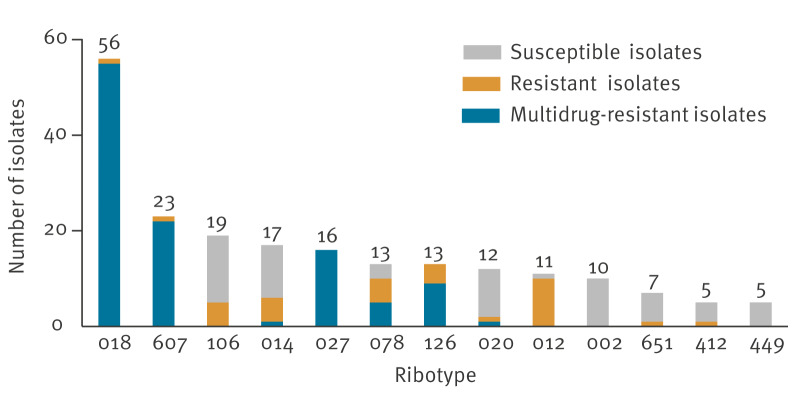
Antimicrobial susceptibility of *Clostridioides difficile* isolates in a pilot study of *Clostridioides difficile* infection in hospitals, by PCR ribotype (RT), Italy, September–December 2022 (n = 270)^a^

^a^ PCR ribotypes present in ≥ 5 isolates included in the figure, i.e. 207 of 270 culturable isolates.

In total, 294 *C. difficile* isolates were sent to the ISS, and 270 (91.8%) of them were culturable on selective plates. A total of 55 RTs were identified, 13 RTs in 207 (76.6%) of the culturable isolates: RT 018 (n = 56; 20.7%), RT 607 (n = 23; 8.5%), RT 106 (n = 19; 7.0%), RT 014 (n = 17; 6.3%), RT 027 (n = 16; 5.9%), RT 078 (n = 13; 4.8%), RT 126 (n = 13; 4.8%), RT 020 (n = 12; 4.4%), RT 012 (n = 11; 4.1%), RT 002 (n = 10; 3.7%), RT 651 (n = 7; 2.6%), RT 412 (n = 5; 1.9%) and RT 449 (n = 5; 1.9%) (Figure 5). Ribotype 607 was more common in patients aged ≥ 65 years (p = 0.0017) and hospitalised in the northern regions of Italy (p < 0.0001), while RT 018 was more common in the central regions (p < 0.0001). More details can be seen in Supplementary Table S2.

Multidrug resistance was defined as resistance to ≥ 3 antimicrobial classes.

Of the 270 *C. difficile* isolates, 267 (98.9%) showed the *tcdA* and the *tcdB* genes and 51 (18.9%) had the CDT genes. Only two isolates were non-toxigenic. More detailed results are presented in Supplementary Table S2.

### Antimicrobial susceptibility

Most (n = 158; 58.5%) *C. difficile* isolates were resistant to at least one antimicrobial class tested, and 119 (75.3 %) of the 158 resistant isolates were MDR (Figure 5). Resistance to ERY, CLI, MXF and/or RIF was most common, as presented in Supplementary Table S2. None of the *C. difficile* isolates had an MTZ or a VAN resistant phenotype.

Isolates of *C. difficile* with the *tcdA* and *tcdB* toxin genes were more frequently associated with a MDR (p < 0.0001) or resistant (p < 0.0001) phenotype. In addition, isolates belonging to RT 018, RT 607 and RT 027 were significantly associated with MDR (p < 0.0001) or resistance to antimicrobials (RT 018 and RT 607: p < 0.0001; RT 027: p = 0.0003).

## Discussion

The present pilot study on CDI surveillance, performed in Italy between September and December 2022, with the participation of hospitals of different levels and geographic locations, provided an overview of CDI in the country, validating an operational protocol for an Italian national CDI surveillance. In general, *C. difficile* testing rate was higher in the primary hospitals than in the other hospital levels, while the percentage of CDI positivity showed slight variations among the hospital levels. The mean overall testing rate of *C. difficile* (55.3 tests/10,000 pd; range: 0–119 tests/10,000 pd) was lower than the testing rate in the EU/EEA countries between 2016 and 2017 (66.1 tests/10,000 pd), while the detection rate of 9.7% (range: 3.0–30. 4%) was higher than the one reported in the EU/EEA survey (2.4%) [9]. The European results were partially affected by the low *C. difficile* testing rate of many European hospitals and the high testing rate reported in the UK (over seven times the rates reported by other countries in the EU/EEA) [9].

In our study, we found a positive correlation between the number of CDI tests performed and the number of CDI cases. The diagnostic algorithms recommended by the ESCMID [15] were used by nine (one primary, four secondary and four tertiary hospital) of the 25 participating hospitals. These hospitals were located in four Italian regions and had a higher proportion of CDI than those using other algorithms, which may indicate a potential sub-optimal CDI diagnosis when an inappropriate diagnostic path is used, as highlighted by previous studies [21,22].

The mean overall rate of CDI (5.0 cases/10,000 pd) was higher than the mean rate in the EU/EEA countries 2016–2017 (3.43 cases/10,000 pd, range: 1.20–12.26 cases/10,000 pd) and almost doubled the Italian value in the European study (2.97 cases/10,000 pd), although only two Italian hospitals participated in that study [9]. In particular, the mean overall rates of both the HA-CDI (3.7 cases/10,000 pd) and the recurrent CDI (0.5 cases/10,000 pd) were higher than the corresponding rates in the EU/EEA countries 2016–2017 (2.34 cases/10,000 pd, range: 0.58–9.26 cases/10,000 pd and 0.29 cases/10,000 pd, range: 0.07–1.98 cases/10,000 pd, respectively), and to the Italian rates reported in those years (2.26 and 0.21/10,000 pd, respectively) [9]. However, the mean overall CA/UA-CDI rate of 0.8 cases/10,000 pd was lower than the mean rate in the EU/EEA countries 2016–2017 (1.15 cases/10,000 pd, range: 0–2.11 cases/10,000 pd), but higher than the rate of 0.5 cases per 10,000 pd reported for Italy in the same period [9].

Higher rates of overall CDI, HA-CDI and CA/UA-CDI were reported in both the secondary and tertiary hospitals compared with the primary hospitals in the present pilot study, while the recurrent CDI rates showed slight variations among hospitals of different levels. These higher values may be in part explained by an increase in the population at higher risk, in particular persons aged > 80 years that are frequently hospitalised and patients with more severe clinical conditions, the use of more appropriate *C. difficile* diagnostic algorithms and more targeted request for CDI testing by clinicians in the secondary and tertiary hospitals, unreported CDI outbreaks during the study period and a general increased care burden (understaffing or overcrowding).

Most cases were detected in the general medicine (36%), surgery (11%) and geriatric (7%) wards. In the ECDC Annual Epidemiological Report for 2016–2017, 72% of patients with CDI were aged ≥ 65 years and 56.4% were female [9]. Likewise, 76.7% of patients with CDI in our study were aged ≥ 65 years and 53.6% were females. Italy has the oldest population in Europe and the second oldest in the world, after Japan (https://www.cia.gov/the-world-factbook/field/median-age/country-comparison/) which may explain the high proportion of patients with CDI aged ≥ 65 years. In our study, 3.3% of deaths were definitely or possibly associated with CDI which was slightly lower than in the EU/EEA countries 2016–2017 (4.6%) [9]. In general, immunosuppression, chronic diseases (e.g., diabetes mellitus, chronic obstructive pulmonary disease or heart failure), functional dysfunction and concurrent use of several medications represent factors that affect the prognosis of geriatric patients. Therefore, it is possible that in a country with many older patients, as Italy, these factors could be the main cause of death. However, we could not fully follow-up the patients because we did not have access to the death registry.

Molecular typing of the 270 isolates showed a heterogeneity of circulating *C. difficile* PCR ribotypes, with 55 RTs identified. The two most common RTs in our study, RT 018 (20.7%) and RT 607 (8.5%), are genetically related and belong to the same lineage [23]. In the EU/EEA countries 2016–2017, only 0.7% of the isolates were RT 018 and none was RT 607 [9]. In our study, 7.0% of isolates belonged to RT 106, while in the EU/EEA countries 2016–2017, only 2.0% of isolates were reported as RT 106. Differently, the percentages of isolates RT 027 and RT 078 were lower in our study (5.9% and 4.8%, respectively) compared to those reported in the EU/EEA countries 2016–2017 (8.1% and 6.8%, respectively)). Similarly, RT 002, the second most common RT in the EU/EEA countries 2016–2017 (8.4%), was rarer in our study (3.7%). Notably, RT 014 and RT 020, singularly considered in this pilot study (6.2% and 4.4%, respectively), were considered together in the ECDC report 2016–2017 [9], and the RT 014/020 were most common in the EU/EEA countries in that period (16.8%) [9].

A different geographic distribution of the two most common RTs was observed, with RT 018 more frequently isolated from the hospitals of the central area of Italy (p < 0.0001) and RT 607 from the hospitals of the northern area of the country (p < 0.0001).

Due to RT 018 and RT 607, positive for toxins A and B but negative for the binary toxin CDT, the proportion of CDT-positive *C. difficile* isolates in the pilot study was lower than in the EU/EEA countries 2016–2017 (19% vs 61.1%) [9].

More than half of the *C. difficile* isolates were phenotypically resistant to at least one antimicrobial class. Interestingly, 75.3% of these resistant isolates were MDR, with resistance patterns including ERY, CLI, MXF and/or RIF.

Resistance to the first-line antimicrobials for the treatment of CDI, metronidazole (MTZ) and vancomycin (VAN), was not observed in this study, while 4.7% of the isolates in the EU/EEA countries 2016–2017 were MTZ-resistant. These MTZ-resistant isolates belonged to RT 027 (76.9%) and RT 036/198 (19.2%), both of the same genetic lineage [9]. Resistance to VAN has not been reported in the EU/EEA countries, except for one suspected VAN-resistant isolate in the ECDC study 2016–2017 which was not confirmed as resistant at the ECDIS-Net-2 reference laboratory (LUMC, the Netherlands) [9].

Our study has some limitations: short time period covered, few regions or autonomous provinces participating, non-randomised sampling strategy and failing to collect all metadata from all participating hospitals. Consequently, the results obtained in this pilot study cannot be generalised to the total hospitalised population in Italy.

## Conclusion

*Clostridioides difficile* infection is associated with high morbidity and mortality. In the Italian hospitals, the mean overall CDI and HA-CDI rates were higher than those reported in a previous study in the EU/EEA countries and highly virulent *C. difficile* types multidrug-resistant to antimicrobials, in particular the genetically related RT 018 and RT 607, were circulating in the hospitals. Therefore, it will be necessary to raise awareness among Italian clinicians, microbiologists and hospital managements, particularly of primary hospitals, on the importance of a correct CDI diagnostic process by training programmes including online courses, workshops and dissemination of recommendations on CDI prevention and diagnosis. The present pilot shows the possibility to build an epidemiological and microbiological CDI surveillance system in Italy to more effectively control and prevent this infection.

## Supplementary Data

277000 Supplementary Material
