# Supplementary material for: Pilot study of Clostridioides difficile infection (CDI) in hospitals, Italy, September to December 2022
Source: Euro Surveill. 2025 Jan 9;30(1):2400206. doi: 10.2807/1560-7917.ES.2025.30.1.2400206 (PMC11719802; doi:10.2807/1560-7917.ES.2025.30.1.2400206)
Supplement: Supplementary Material [file 24-00206_SPIGAGLIA_Supplement.pdf]

"This supplementary material is hosted by Eurosurveillance as supporting information alongside the article "Pilot study of Clostridioides difficile infection (CDI) in hospitals, Italy, September to December 2022", on behalf of the authors, who remain responsible for the accuracy and appropriateness of the content. The same standards for ethics, copyright, attributions and permissions as for the article apply. Supplements are not edited by Eurosurveillance, and the journal is not responsible for the maintenance of any links or email addresses provided therein."

Criteria of inclusion for hospitals, wards and patients, and definitions for CDI case, new CDI case, CDI recurrence, origin of infection (healthcare associated CDI - HA-CDI, community associated CDI - CA-CDI, and unknown associated CDI - UA-CDI), used in the pilot study referred to: European Centre for Disease Prevention and Control. European Surveillance of *Clostridioides* (*Clostridium*) *difficile* infections. Surveillance protocol version 2.4. Stockholm: ECDC; 2019.

Hospitals: All hospital levels were included in the study. The Italian hospital levels are three: primary (catchment area of 80 000 - 150 000 inhabitants), secondary (catchment area of 150 000 - 300 000 inhabitants, with an emergency department and specialties), and tertiary (catchment area of 600 000 – 1 200 000 inhabitants, with an emergency department and many specialties, often university hospitals or associated with a university).

Wards: All wards were included in the study, except for day cases (e.g. one-day surgery, patients in the emergency room without an overnight stay, dialysis patients) that were not included.

Patients: All hospitalized patients were included in the study, but children aged two years or less were included only if significant clinical evidence of CDI was observed. To be included in the study, all hospitalized patients with CDI met at least one of the following criteria: i) the onset date of CDI symptoms was within the surveillance period (even if the patient was hospitalized before starting surveillance), or ii) the patient was admitted to the hospital during the surveillance period and had symptoms at the time of admission, even if CDI was diagnosed prior to admission, or iii) in case of recurrent CDI.

CDI cases: CDI cases included in the study met the following criteria: i) diarrhoeal stools or toxic megacolon and a positive laboratory analysis for the presence of *C. difficile* toxin A and/or B in the stool specimen or identification of toxin-producing *C. difficile* in the stool specimen, or ii) pseudomembranous colitis demonstrated by endoscopic examination of the lower gastrointestinal tract, or iii) colonic histopathology characteristic of *C. difficile* infection (with or without diarrhoea) on a specimen obtained during endoscopy, colectomy, or autopsy.

New CDI cases: Defined as cases with a positive laboratory CDI test after two weeks from the disappearance of symptoms of a previous CDI episode or, in the absence of the date of disappearance of symptoms, after two weeks from the last positive stool sample for CDI, were included in the surveillance study. Patients with a recurrence, defined as a CDI case with a positive *C. difficile* specimen between two and eight weeks after the disappearance of the symptoms of the previous episode or, in the absence of the date of disappearance of the symptoms, between two and eight weeks after the last positive faecal sample for CDI, were also included in the study.

Recurrences: Since in the clinic it is not possible to differentiate between a relapse, in which the infection is caused by the same strain of *C. difficile*, and a re-infection, caused by a different strain,

the term recurrence was used to define both circumstances. When a positive laboratory test for CDI was obtained within 14 days of the disappearance of symptoms of a previous CDI episode or, in the absence of the date of disappearance of symptoms, within 14 days of the last positive stool sample for *C. difficile*, this positivity was not considered a new CDI case and therefore it was not included in the surveillance study.

Origin of CDI: The origin of a CDI case was based on the location and date of onset of symptoms. If the date of onset was not available, the date of the first *C. difficile* positive diagnostic test was considered. According to the origin, the cases was classified as healthcare-associated CDI (HA-CDI) if the onset of CDI symptoms occurred i) 48 hours after admission to a care facility, or ii) within the first 48 hours of admission to a care facility but within four weeks of discharge from a care facility (previous admission to the same facility or another hospital or social-healthcare facility). Differently, a case was considered community-associated CDI (CA-CDI) when onset of symptoms occurred i) outside of healthcare facilities and without discharge of patient from a healthcare facility within the previous 12 weeks, or ii) within 48 hours of admission to a healthcare facility in a patient that was not resident in a healthcare facility within the previous 12 weeks. Finally, a case was defined unknown association CDI (UA-CDI) when onset of symptoms occurred i) outside of healthcare facilities in a patient that was discharged from a healthcare facility within the previous 4–12 weeks, or ii) within 48 hours of admission to a healthcare facility in a patient that was resident in a healthcare facility within the previous 4–12 weeks.

Table S1: Diagnostic algorithms, *C. difficile* positivity, CDI incidence and MDR isolates according to the hospital levels of the different Italian regions/autonomous provinces participating in the CDI surveillance pilot study; Italy, September - December 2022.

Region or Autonomous province	Hospital level	CD[a] diagnostic algorithm (N. of hospitals)	Overall percentage of CD positive tests	Overall CDI incidence [c] (N. of cases/10 000 pd)	Total N. of isolates	MDR[e] isolates (%)	PCR-ribotype of MDR isolates (N.)
**Lombardia**	Secondary	GDH and Tox A/B EIA + NAAT or TC (1) [b]	3.0%	5.7	32	18 (56.2%)	027 (5) 126 (5) 018 (3) 607 (3) 078 (2)
	Tertiary	GDH EIA + NAAT or TC (2)	8.5%	nd [d]	44	9 (20.5%)	018 (2) 027 (2) 020 (1) 078 (1) 126 (1) 181 (1) 607 (1)
		NAAT (1)	7.9%	2.7	18	7 (38.9%)	027 (4) 078 (1) 126 (1) 607 (1)
**Autonomous province of Trento**	Secondary	NAAT (1)	12.1%	6.0	0	0	
**Veneto**	Primary	GDH EIA + NAAT or TC (2)	7.8%	3.1	0	0	
		Unknown (6)	6.9%	1.8	11	4 (36.4%)	607 (4)
	Secondary	GDH and Tox A/B EIA + NAAT or TC (1) [a]	14.1%	4.3	0	0	
		NAAT + Tox A/B EIA (1) [a]	30.4%	11.9	10	3 (30.0%)	607 (2) 181 (1)
		GDH EIA + NAAT or TC (1)	13.5%	nd	0	0	
	Tertiary	GDH and Tox A/B EIA + NAAT or TC (2) [a]	7.0%	4.1	26	14 (53.9%)	607 (11) PR31977 (2) 018 (1)
**Umbria**	Secondary	Tox A/B EIA + NAAT or TC (1)	11.3%	6.9	39	9 (23.1%)	018 (6) 014 (1) 027 (4) 126 (1)
**Lazio**	Primary	GDH and Tox A/B EIA + NAAT or TC (1) [a]	12.1%	7.5	9	4 (44.4%)	018 (2) 047 (1) 078 (1)
	Secondary	GDH and Tox A/B EIA + NAAT or TC (1) [a]	14.6%	11	23	18 (78.3%)	018 (16) 039 (1) PR31428 (1)
	Tertiary	GDH and Tox A/B EIA + NAAT or TC (1) [a]	17.0%	7.6	16	13 (81.2%)	018 (9) 027 (2) 181 (1) PR31428 (1)
		NAAT if GDH EIA positive and Tox A/B EIA negative (1)	14.5%	3.5	28	15 (53.6%)	018 (11) 027 (2) 126 (1) 181 (1)
**Puglia**	Tertiary	GDH EIA (1)	8.9%	1.7	10	5 (50.0%)	018 (5)
**Sicilia**	Tertiary	GDH and Tox A/B EIA + NAAT or TC (1) [a]	9.3%	6.8	4	0	

[a] CD: *Clostridioides difficile*; [b] ESCMID-recommended algorithms; [c] Data reported by 21/25 hospitals participating in the pilot study; [d] nd: not determined; [e] MDR: multi resistant to antimicrobials

Table S2: Toxigenic profiles and resistance patterns of the 270 strains isolated in the CDI surveillance pilot study; Italy, September - December 2022.

PCR-ribotype	Number of strains (%)	Toxigenic profile [a]	Phenotypic resistance patterns[b] (number of strains)
001	2 (0.7%)	A+ / B+ / CDT-	S (2)
002	10 (3.7%)	A+ / B+ / CDT-	S (10)
005	3 (1.1%)	A+ / B+ / CDT-	S (2), ERY CLI (1)
010	1 (0.4%)	A- / B- / CDT-	S (1)
012	11 (4.1%)	A+ / B+ / CDT-	ERY CLI (10), S (1)
014	17 (6.3%)	A+ / B+ / CDT-	S (11), MXF (2), RIF (2), ERY CLI (1), MXF ERY CLI (1)
015	1 (0.4%)	A+ / B+ / CDT-	S (1)
018	56 (20.7%)	A+ / B+ / CDT-	MXF ERY CLI RIF (54), ERY CLI RIF (1), MXF (1)
020	12 (4.4%)	A+ / B+ / CDT-	S (10), MXF ERY CLI RIF (1), MXF (1)
023	2 (0.7%)	A+ / B+ / CDT+	S (2)
027	16 (5.9%)	A+ / B+ / CDT+	MXF ERY CLI RIF (15), MXF ERY CLI (1)
029	1 (0.4%)	A+ / B+ / CDT-	S (1)
039	1 (0.4%)	A- / B- / CDT-	MXF ERY CLI (1)
043	1 (0.4%)	A+ / B+ / CDT-	S (1)
046	1 (0.4%)	A+ / B+ / CDT-	MXF RIF (1)
047	1 (0.4%)	A+ / B+ / CDT-	MXF ERY CLI RIF (1)
049	1 (0.4%)	A+ / B+ / CDT-	S (1)
053	1 (0.4%)	A+ / B+ / CDT-	S (1)
054	3 (1.1%)	A+ / B+ / CDT-	S (3)
056	2 (0.7%)	A+ / B+ / CDT-	S (2)
070	4 (1.5%)	A+ / B+ / CDT-	S (4)
076	3 (1.1%)	A+ / B+ / CDT-	S (3)
078	13 (4.8%)	A+ / B+ / CDT+	MXF ERY CLI (5), ERY CLI (4), MXF RIF (1), S (3)
083	1 (0.4%)	A+ / B+ / CDT-	S (1)
087	3 (1.1%)	A+ / B+ / CDT+	S (3)
106	19 (7.0%)	A+ / B+ / CDT-	S (14), ERY CLI (2), MXF (2), RIF (1)
126	13 (4.8%)	A+ / B+ / CDT+	MXF ERY CLI (8), MXF ERY CLI RIF (1), ERY CLI (4)
181	4 (1.5%)	A+ / B+ / CDT+	MXF ERY CLI RIF (4)
211	1 (0.4%)	A+ / B+ / CDT-	S (1)
220	2 (0.7%)	A+ / B+ / CDT-	ERY CLI (2)
236	1 (0.4%)	A+ / B+ / CDT-	S (1)
241	1 (0.4%)	A+ / B+ / CDT-	S (1)
242	1 (0.4%)	A+ / B+ / CDT+	S (1)
412	5 (1.9%)	A+ / B+ / CDT-	S (4), ERY CLY (1)
425	1 (0.4%)	A+ / B+ / CDT-	S (1)
427	1 (0.4%)	A+ / B+ / CDT+	S (1)
449	5 (1.9%)	A+ / B+ / CDT-	S (5)
464	2 (0.7%)	A+ / B+ / CDT-	S (2)
471	1 (0.4%)	A+ / B+ / CDT+	S (1)
607	23 (8.5%)	A+ / B+ / CDT-	MXF ERY CLI RIF (22), RIF (1)

651	7 (2.6%)	A+ / B+ / CDT-	S (6), ERY CLY (1)
712	1 (0.4%)	A+ / B+ / CDT-	S (1)
742	1 (0.4%)	A+ / B+ / CDT-	S (1)
017/1	1 (0.4%)	A+ / B+ / CDT-	RIF (1)
033/1	1 (0.4%)	A+ / B+ / CDT+	S (1)
AI-21/0	1 (0.4%)	A+ / B+ / CDT-	S (1)
AI-58	1 (0.4%)	A+ / B+ / CDT-	S (1)
AI-60	1 (0.4%)	A+ / B+ / CDT-	S (1)
AI-82/1	1 (0.4%)	A+ / B+ / CDT-	S (1)
AI-84	1 (0.4%)	A+ / B+ / CDT-	S (1)
PR31428	2 (0.7%)	A+ / B+ / CDT-	MXF ERY CLI RIF (2)
PR31959	1 (0.4%)	A+ / B+ / CDT-	S (1)
PR31977	2 (0.7%)	A+ / B+ / CDT-	MXF ERY CLI RIF (2)
PR32207	1 (0.4%)	A+ / B+ / CDT-	S (1)
PR32401	1 (0.4%)	A+ / B+ / CDT-	S (1)

[a] A: Toxin A; B: Toxin B; CDT: Binary toxin CDT
[b] ERY: erythromycin; CLI: clindamycin; MXF: moxifloxacin; RIF: rifampicin; S: susceptible to the antimicrobials tested
